# Pharmacological, Neurochemical, and Behavioral Mechanisms Underlying the Anxiolytic- and Antidepressant-like Effects of Flavonoid Chrysin

**DOI:** 10.3390/molecules27113551

**Published:** 2022-05-31

**Authors:** Juan Francisco Rodríguez-Landa, León Jesús German-Ponciano, Abraham Puga-Olguín, Oscar Jerónimo Olmos-Vázquez

**Affiliations:** 1 Laboratorio de Neurofarmacología, Instituto de Neuroetología, Universidad Veracruzana, Xalapa 91190, Mexico; lgerman@uv.mx (L.J.G.-P.); oscarplsstahp1@gmail.com (O.J.O.-V.); 2 Facultad de Química Farmacéutica Biológica, Universidad Veracruzana, Xalapa 91000, Mexico; 3 Unidad de Salud Integrativa, Centro de EcoAlfabetización y Diálogo de Saberes, Universidad Veracruzana, Xalapa 91060, Mexico; abpuga@uv.mx

**Keywords:** antidepressant, anxiolytic, chrysin, flavonoid, natural medicine, neuropharmacology

## Abstract

Chrysin (5,7-dihydroxyflavone) is a flavonoid isolated from plants, such as *Passiflora coerulea*, *Passiflora incarnata*, and *Matricaria chamomilla*. This natural molecule exerts diverse pharmacological effects, which includes antioxidant, anti-inflammatory, anti-cancer, neuroprotective, and anti-apoptotic effects. Additionally, in brain structures, such as the hippocampus, prefrontal cortex, raphe nucleus, and striatum, involved in the physiopathology of anxiety and depression disorders, several neuropharmacological activities, including the activation of neurotransmitter systems (GABAergic, serotonergic, dopaminergic, and noradrenergic), neurotrophic factors, such as brain-derived neurotrophic factor and the nerve growth factor, and some signaling pathways are affected. The results showed that the anxiolytic and antidepressant-like effects of chrysin occurs through its interaction with specific neurotransmitter systems, principally the GABAergic and the serotonergic, and activation of other neurotrophic factors. However, it is not possible to discard the antioxidant and anti-inflammatory activities of chrysin while producing its anxiolytic- and antidepressant-like effects. Although these results have been obtained principally from pre-clinical research, they consistently demonstrate the potential therapeutic use of flavonoid chrysin as an anxiolytic and antidepressant agent. Therefore, this flavonoid could be considered as a promising novel therapy for anxiety and depression disorders.

## 1. Introduction

Throughout the development of pharmacology, the study of natural molecules has been of special importance to identify secondary metabolites from plants and to stimulate the discovery and design of new drugs for the treatment of diseases in humans [1]. The multidisciplinary study of diverse molecules extracted from plants, isolated, and chemically characterized, has permitted the identification of the mechanism of action involved in its beneficial and toxic effects [2]. The use of molecular, biochemical, pharmacological, histological, and behavioral techniques has helped develop multi-target drugs for the prevention and treatment of diverse diseases, particularly those that have recently increased in association with social dynamics, lifestyle, and environmental factors, such as neuropsychiatric and stress-related disorders [3].

Polyphenols are a group of molecules with multiple beneficial effects on health. Its antioxidant effects beneficially impact the physiological process of the organism [4]. Among polyphenols, flavonoids have been widely studied to develop complementary therapeutic strategies for the treatment of metabolic, cardiovascular, and neuropsychiatric disorders and cancer [5,6,7]. In vitro and in vivo studies on the pharmacological action of the flavonoid chrysin have identified multiple effects of it on different systems in the organism [8,9,10], including the central nervous system (CNS). Chrysin exerts a neuropharmacological effect in brain structures such as the amygdala, hippocampus (HP), prefrontal cortex (PFC), and raphe nucleus, which are involved in the physiopathology of several neuropsychiatric disorders, such as anxiety and depression [11,12,13,14]. The effects include the activation of the GABAergic system by modulating the GABA_A_/benzodiazepine receptor complex [14,15,16,17]; changes in serotonin levels and modification in the expression of their receptors, such as 5-HT_1A_ and 5-HT_2A_ in the raphe nucleus and HP [13,18]; and restoration of dopamine and noradrenaline levels in the CNS [12,19]. In addition, chrysin activates neurotrophic factors that increase brain-derived neurotrophic factor (BDNF) and nerve growth factor (NGF) levels, thereby activating signaling pathways in the brain [11,20]. However, anti-inflammatory, anti-apoptotic, and neuroprotective effects should be considered as potential mechanisms of action involved in their anxiolytic- and antidepressant-like effects, considering that neuroinflammation and apoptotic processes are involved in the pathophysiology of anxiety and depression disorders [21].

In this review, we describe, analyze, and discuss the scientific results from pre-clinical research that report the pharmacological, neurochemical, and behavioral mechanisms that could underlie the anxiolytic and antidepressant effects of flavonoid chrysin, which is considered as a promising therapy for anxiety and depression disorders. In addition, we propose the incorporation of chrysin as a potential anxiolytic and antidepressant drug in future scientific research, not only for its classical actions on neurotransmission systems, but also for its antioxidant and anti-inflammatory effects, and the activation of neurotrophic factors and the associated signaling pathways. This may contribute to the development of specific therapies for anxiety and depression disorders according to their etiology.

## 2. Generalities of the Flavonoid Chrysin

Flavonoids are polyphenolic compounds that are present in plants. They produce pharmacological actions in the peripheral and CNS [9]. They can cross the blood–brain barrier and interact with several neurotransmission systems and, thereby activating signaling pathways in specific brain structures involved in the physiopathology of anxiety and depression disorders [10]. In particular, the flavonoid chrysin (5,7-dihydroxyflavone) has been studied for its antioxidant properties; however, its neuropharmacological effects in specific brain structures involved in the physiopathology of several neuropsychiatric disorders, such as anxiety and depression, need to be studied [9,10,14].

**Figure 1:**
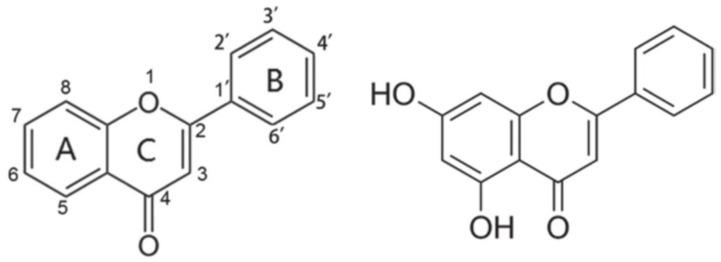
Basic structure of flavones showing fused A and C rings, and phenyl B rings with corresponding numbering system (**left figure**). Structure of the flavonoid chrysin, 5,7-dihydroxyflavone (**right figure**).

Chrysin (Figure 1) has a backbone structure that consists of a fused A and C rings, and a phenyl B ring, which is attached to the second position of ring C and shares the basic structure of the flavones, with an additional hydroxyl group at the fifth and seventh positions of the A ring. The potential of chrysin to act as a free radical scavenger has been attributed to the presence of these hydroxyl groups [20,22], and it has been suggested that these functional groups represent the main site of action of this flavonoid to produce a great variety of pharmacological activities and therapeutic effects. It has the potential to be used as an alternative in the treatment of metabolic, cardiovascular, and neuropsychiatric disorders [10]. In addition, the presence of hydroxyl groups in the backbone of chrysin has been associated with its anxiolytic-like effects [23]. Chrysin, but not the flavone backbone, decreases anxiety-like behavior in rats and zebrafish, suggesting that the presence of hydroxyl groups in its basic structure is indispensable for producing anxiolytic-like effects in pre-clinical research [23].

Chrysin, either isolated from plants like *Passiflora coerulea*, *Passiflora incarnata*, and *Matricaria chamomilla*, or even as a synthetic drug, produces anxiolytic- and antidepressant-like effects. These effects involve several activations of neurotransmission systems and signaling pathways, including the serotonergic and GABAergic systems, and the activation of neurotrophic factors, such as BDNF and NGF. It is likely that the activation of anti-inflammatory and antioxidant signaling pathways may also be involved in these effects. Although these effects have been principally evaluated in pre-clinical research [14,16,17,18,24,25,26], they show the potential therapeutic use of chrysin for anxiety, depression, and other neuropsychiatric disorders.

## 3. Biochemical and Pharmacological Activity of Flavonoid Chrysin

Diverse flavonoids, including chrysin, exert significant anxiolytic- and antidepressant-like activities in mammals and non-mammals, similar to those produced by clinically effective anxiolytic and antidepressant drugs [5,10,13]. These effects have been related to its neurochemical activity on the CNS that impact neuronal functioning (Table 1).

### 3.1. Action of Chrysin on Neurotransmission Systems

Chrysin was the first monoflavonoid reported as a specific ligand for central and peripheral benzodiazepine-binding sites [15]. It was identified when chrysin inhibited the [^3^H] flunitrazepam binding in synaptosomal membranes of the bovine cerebral cortex. Similarly, chrysin also displaced the binding of [^3^H]Ro 5-4864, a potent ligand for the peripheral benzodiazepine receptor, to kidney membranes in a mixed, competitive and non-competitive manner. Interestingly, intracerebroventricular microinjection of chrysin prevents tonic-clonic seizures induced by pentylenetetrazol [15]. This effect was blocked by Ro 15-1788, a central benzodiazepine receptor antagonist, suggesting that this flavonoid exerts some of its pharmacological actions on the benzodiazepine-binding site of the GABA_A_ receptor, enhancing the activation of GABAergic neurotransmission [15]. In support of this, in vitro studies have reported that chrysin and other flavonoids modulate the chloride ion channel in the GABA_A_ receptor in a manner different from benzodiazepines [35].

Additionally, chrysin can also activate other neurotransmitter systems involved in the physiopathology of several neuropsychiatric disorders, including anxiety and depression. Chrysin at 10 mg/kg for 28 days restored dopamine, homovanillic acid, and 3,4-dihydroxyphenylacetic acid levels in the stratum of male C57B/6J mice, in which a depletion of this neurotransmitter was induced by 6-hidroxidopamine [19]. In addition, chrysin at 50, 100, and 200 mg/kg for 5 days prevented the decrease in dopamine in the striatum produced by the injection of 1-methyl-a,2,3,6-tetrahidropidine in C57BL/6J mice [40]. In contrast, chrysin at 20 mg/kg for 28 days reversed the decrease in dopamine and serotonin levels in the PFC and HP of female mice subjected to a hypothyroidism model [12]. Chrysin at 50 mg/kg for 4 days and chrysin at 3–30 mg/kg for 2 weeks significantly increased serotonin levels in mice and rat brains [38,39], while chrysin at 50, 100, and 150 mg/kg attenuated the increased noradrenaline and corticosterone levels in male Wistar rats with pain induced by formalin [41].

It has been shown that the flavonoid chrysin has a positive impact on several neurotransmitter systems that participate in the etiology of anxiety and depression; however, it is also capable of modifying several signaling pathways and activating neurotrophic processes in the CNS, which could be related to its anxiolytic- and antidepressant-like effects.

### 3.2. Antioxidant Activity of Chrysin

Psychiatric disorders have been linked to alterations in the activity of antioxidant enzymes in the CNS, leading to an increase in the production of reactive oxygen species (ROS) and, in the first instance, induction of oxidative stress processes [44]. Oxidative stress leads to an imbalance between the level of antioxidants and the production of ROS, which can generate long-term alterations, such as damage to neuronal membranes with potential activation of apoptosis processes associated with the increase of ROS [45]. These alterations, induced by oxidative stress, are involved in the etiology of anxiety and depression disorders [46,47]. Chrysin has the capacity to modulate the nitric oxide (NO) pathway, which contributes to the production of ROS, in both in vivo and in vitro assays [31,33]. In a diabetic rat model, chrysin at 60 mg/kg for 28 days inhibited oxidative stress by restoring the alterations in NO, glutathione (GSH), catalase (CAT), thiobarbituric acid reactive substances (TBARS) levels, along with the restoration of the activity of superoxide dismutase (SOD), nitrotyrosine (NT), and NADPH oxidase 4 (Nox4) [29]. The same effect was produced by the administration of chrysin at 20 mg/kg for 30 days, exerting a reduction in ethanol-induced toxicity, as evidenced by decreasing markers of oxidative stress in organs such as the liver, kidney, and heart of rats [22]. In addition, chrysin at 50 mg/kg restored the levels of oxidative stress markers, such as GSH, malondialdehyde (MDA), CAT, SOD, and ROS under doxorubicin-induced acute cardiotoxicity in rats, to normal values [27]. The antioxidant effects of chrysin described above occur in the peripheral organs of metabolically challenged (diabetic) or chemically treated (ethanol and doxorubicin) animals; which is important because diabetes and alcohol intake is associated with oxidative stress and inflammation [48,49], and may predispose to anxiety and depression disorders [50,51]. Interestingly, it has been previously reported in preclinical and clinical studies that reduced oxidative stress at the peripheral level is associated with a decrease in anxiety and depression symptoms [52,53,54,55]. In this way, it is possible that peripheral and central antioxidant effects of chrysin could contribute to its anxiolytic and antidepressant effects; however, this aspect needs to be explored further.

In support, daily administration of chrysin at 1 and 10 mg/kg for 60 days restored the activity of SOD and CAT in the HP and PFC of aged mice and reduced oxidative stress by decreasing ROS and inversely increasing BDNF levels in these brain structures [20]. These last data are important because a reduction in anxiety-like behaviors in the elevated plus maze (EPM) test was related to high levels of BDNF, which was accompanied by greater expression of TrkB and BDNF *m*RNA in brain structures such as the HP, PFC, and amygdala [56]. Interestingly, the anxiolytic-like activity produced by diazepam has been associated with the restoration of normal levels of antioxidant enzymes, such as SOD and CAT, as well as a reduction in nitrite concentration in the mouse brain [57]. Therefore, alterations in peripheral and central enzymatic antioxidant systems can generate oxidative stress and contribute to anxiety- and depression-like behaviors. In this way, it is possible that these alterations related to oxidative stress may be restored by the flavonoid chrysin and contribute to its anxiolytic- and antidepressant-like effects, without discarding the participation of other neurochemical processes in the CNS.

### 3.3. Anti-Inflammatory and Anti-Apoptotic Activity of Chrysin

In vitro studies with chrysin showed attenuated release of pro-inflammatory mediators by inhibiting prostaglandins E2 (PGE2) and tumor necrosis factor-α (TNF-α) from lipopolysaccharide-induced RAW 264.7 cells [31]. Chrysin regulates inflammatory and apoptotic signaling pathways by attenuating the effects of nuclear factor-κB (NF-κB) expression, inhibition of nuclear factor kappa-B kinase (IKK-β), TNF-α, interleukin-1β (IL-1β), interleukin-6 (IL-6), and cycloxygenase-2 (COX-2) [27,28,32]. In addition, in a mouse model of polymicrobial sepsis induced by cecal ligation, the administration of chrysin at 5 mg/kg reduced TNF-α levels [33]. In addition, chrysin at 50 and 60 mg/kg for 12 to 28 days in models of myocardial injury in diabetic rats or doxorubicin-induced acute cardiotoxicity exerts anti-inflammatory effects. These effects were associated with the inhibition of TNF-α and NF-κB/IKK-β expression, as well as with the reduced apoptosis due to an increase in Bcl-2 (anti-apoptotic protein) expression and decrease in the expression of pro-apoptotic markers, such as Bax and caspase-3 [27,28]. These effects of chrysin at the peripheral level and in different models of inflammation support its potential anti-inflammatory and anti-apoptotic activities. This is important because peripheral and central pro-inflammatory processes have been involved in anxiety development and depression disorders [58], while some anxiolytic effects of drugs have been related to stable functioning of COX-2 in the infralimbic and prelimbic cortex, HP, and ventral tegmental area [59]. In addition, anxiolytic- and antidepressant-like effects produced while exercising are due to the increase in Bcl-2 and the reduction of Bax and caspase-3 in the dorsal raphe nucleus of rats evaluated in the EPM and forced swim test (FST) without observing neuronal death [60,61]. In contrast, a reduction in Bcl-2 and an increase in Bax in the frontal cortex are related to anxiety-like behavior and other stress-related disorders [60]. Therefore, chrysin can produce anxiolytic- and antidepressant-like effects by exerting beneficial effects on these biochemical systems.

Similarly, chrysin at 5 and 20 mg/kg for 28 days produced significant changes in neurochemical factors that are implicated in the reduction of pro-inflammatory cytokines in mice subjected to the chronic unpredictable mild stress model (CUMS) [18]. Furthermore, chrysin also prevented the increase in concentrations of corticotropin-releasing hormone (CRH) and adrenocorticotropic hormone (ACTH) in plasma, while the levels of TNF-α, IL-1β, and IL-6 in the PFC and HP were reduced by both doses of chrysin [18]. Similarly, under these conditions, restoration of caspase-3 and caspase-9 activity in the HP and PFC was observed. All these changes were associated with a reduction in depression-like behaviors, reaching values similar to those reported in unstressed mice [18].

It is necessary to highlight that mice and rats displayed lower anxiety-like behavior in the EPM test showing lower expression of pro-inflammatory cytokines such as TNF-α, IL-1β, and IL-6 in the HP, frontal cortex, and serum [44,62]. However, a contrary effect was reported in animals showing high anxiety-like behavior. Additionally, chrysin can regulate the hypothalamic–pituitary–adrenal (HPA) axis and physiological responses to stressful situations [18]. Adequate regulation of the HPA axis combined with the lower production and release of corticosterone has been associated with the adequate coping with stress and low risk of developing neuropsychiatric disorders [63,64].

Altogether, these results suggest that neurochemical changes produced by flavonoid chrysin, in addition to suppressive effects on inflammatory and oxidative processes, could contribute to its anxiolytic- and antidepressant-like effects [42,65]. These results support the idea that chrysin could be considered a potential candidate for ameliorating anxiety and depression symptoms in humans.

### 3.4. Effects of Chrysin on Gut Microbiota

Currently, there is growing evidence proposing that dysregulation in the composition of the gut microbiota is related to the pathophysiology of anxiety and depression disorders due to its interaction with neuroimmune, neuroendocrine, and neural pathways [66,67]. These pathways are part of the brain–gut–microbiota axis that may modulate brain development and function, which also impacts on behavior. For example, a fecal microbiota sample from depressed patients when transferred by oral gavage to a microbiota-deficient rat model produced depressive-like behaviors in the recipient animal [68]. Contrarily, fecal microbiota coming from intact rats transplanted to chronically stressed rats significantly reduced depression- and anxiety-like behaviors and anhedonia, which was associated with the suppressed activation of glial cells and the NLRP3 inflammasome in the brain [69]. Interestingly, polyphenols may regulate the composition of the gut microbiota, promoting beneficial effects on the intestinal microbiota and inhibiting the proliferation of harmful bacteria; thereby maintaining intestinal health and ameliorating some neurological and neuropsychic disorders [70,71]. This is important because a correct structure of gut microbiota in addition to an anti-inflammatory effect of diverse molecules, including polyphenols, has been associated with its antidepressant-like effects [72]. In the specific case of chrysin, its beneficial effects in modulating the structural alteration of the gut microbiota in mice have been explored [73]. Chrysin at 10 mg/kg for 28 days attenuated the increase in pro-inflammatory cytokines and the intestinal damage induced by LPS in C57BL6/J mice, which was associated with adequate gut microbiota structure [74]. Likewise, chrysin can modulate intestinal inflammation in Caco2 cells stimulated with IL-1B, improving intestinal absorption and metabolic stability [75], which could be related with a normal function of the gut microbiota. This is important because anti-inflammatory effects of several molecules at the peripheral and central level have been associated with a reduction of anxiety and depression-like behavior [58]. In this sense, it is possible that the regulation of the gut microbiota by polyphenols such as chrysin may open new perspectives to explore the effects of chrysin on the brain–gut–microbiota axis and its potential relationship with its anxiolytic- and antidepressant-like effects.

## 4. Anxiolytic-like Effects of Flavonoid Chrysin

In 1994, Wolfman et al. reported an anxiolytic-like effect of chrysin in mice. A single dose of chrysin at 1 mg/kg significantly increased the time spent in open arms of the EPM [24]. In the light/dark box (LDB), increased time was spent in the illuminated compartment [17], and in both cases the effects were similar to that produced by diazepam. These behavioral effects are considered to be associated with anxiolytic-like effects in pre-clinical research. Chrysin, but not diazepam, is devoid of motor effects related to sedation [24] and this may represent the advantage of chrysin over benzodiazepines, such as diazepam, in the treatment of anxiety disorders [76]. Interestingly, in male Sprague Dawley rats, the anxiolytic-like effects of chrysin at 1 and 2 mg/kg in LDB were blocked by a previous administration of flumazenil [17,77], an antagonist of the benzodiazepine binding site in the GABA_A_ receptor. Additionally, acute administration of chrysin (1 mg/kg) produced anxiolytic-like effects in male Wistar rats [78] and CD-1 male mice [79] evaluated in the EPM. Similarly, anxiolytic-like effects of chrysin in mammals (mice and rats) have also been reported in non-mammalian organisms (zebrafish). Chrysin at 1 mg/kg decreased anxiety-like behavior in rats and zebrafish, similar to diazepam [23]; however, treatment with a flavone backbone at 1 mg/kg was devoid of anxiolytic-like effects in both rats and zebrafish, suggesting that the presence of hydroxyl groups in its basic structure could be indispensable to produce anxiolytic-like effects [23].

**Figure 2:**
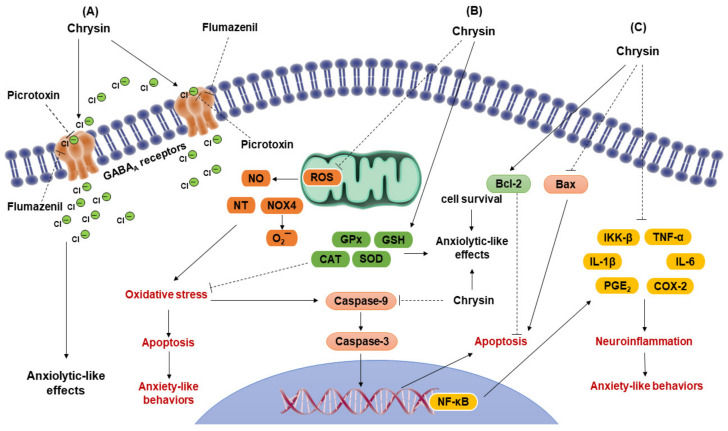
Mechanism of action of the flavonoid chrysin potentially involved in its anxiolytic-like effects. (**A**) It has been confirmed that chrysin produces its anxiolytic-like effect through its action on the GABA_A_/benzodiazepine receptor complex producing configurational changes in the receptor and regulating the opening of the Cl^−^ ion channel [14,15,17,24,35], which may produce inhibitory effects in the GABAergic system associated with its anxiolytic-like effects. These effects can be blocked by specific antagonists of the GABA_A_ receptor, such as picrotoxin, bicuculline, and flumazenil [14]. (**B**) Probably, antioxidant effects of chrysin could be involved in its anxiolytic-like effects. Chrysin significantly reduces ROS by inhibiting the production of NO, NT, and NOX4 [29]. These effects reduce the oxidative stress and reduces the neuronal damage. Additionally, chrysin reduces the activity of Bax, caspase-9, and caspase-3, while increasing the production of Bcl-2, thereby reducing the damage of DNA and inhibiting apoptotic processes [60,61], which reduces the neuronal death. (**C**) Additionally, the anti-inflammatory effects of chrysin could contribute to its anxiolytic-like effects, considering that it may reduce the inflammatory response by inhibiting the signaling pathway NF-κB/IKK-β [27,28]. Chrysin may attenuate the expression of NF-κB that participates as transcriptional factors at nuclear level, binding to genes that induce neuro-inflammation process. Chrysin also inhibits the production of pro-inflammatory cytokines, such as IL-1β and IL-6, in addition to suppressing the production of proinflammatory mediators, such as TNF-α, PGE_2_ and COX-2 [27,28,32]. These effects could reduce neuro-inflammation associated with the anxiety-like behavior. ROS = reactive oxygen species; NO = nitric oxide; NT = nitrotyrosine; NOX4 = NADPH oxidase; O2¯ = superoxide; Green circles = chlorine ions; SOD = superoxide dismutase; GSH = reduced glutathione; CAT = catalase; GPx = glutathione peroxidase; Bcl-2 = anti-apoptotic protein of the subfamily Bcl-2; Bax = pro-apoptotic protein of the subfamily Bax; NF-κB = nuclear factor kappa B; IKK-β = inhibitor of nuclear factor kappa-B; TNF-α = tumor necrosis factor-α; IL-1β = interleukin-1β; IL-6 = interleukin-6; PGE2 = prostaglandins E2; COX-2 = cycloxygenase-2. (Figure was prepared by the authors).

Anxiety symptoms in women are associated with a reduction in steroid hormones, such as estradiol and progesterone, and its reduced metabolite allopregnanolone in the peripheral and CNS, which may occur pre-menstruation, post-partum, and during the transition to menopause stage [80,81]. These steroid hormones may modulate several neurotransmission systems, such as the serotoninergic, noradrenergic, dopaminergic, and GABAergic [82]; therefore, some of these hormones have been proposed as novel groups of anxiolytic drugs for treating particular anxiety and depression disorders associated with reduced concentrations of steroid hormones [83]. It has recently been proposed that the flavonoid chrysin mimics some of the pharmacological effects of neurosteroids in female rats [37]. Anxiety-like behaviors in female rats significantly increase during the metestrus–diestrus phase of the ovarian cycle, which is associated with a low concentration of steroid hormones [84]; this phase is considered an equivalent of the premenstrual period in women [85]. Interestingly, chrysin at 2 mg/kg, similar to diazepam at 2 mg/kg, prevents anxiety-like behavior that naturally occurs during the metestrus–diestrus phase in female rats evaluated in the EPM and LDB. This effect can be blocked by a previous injection of picrotoxin [36]. In support, microinjection of chrysin at 0.5 μg in the dorsal HP prevented anxiety-like behavior that naturally occurs during diestrus, which was blocked by previous injection of picrotoxin, bicuculline, and flumazenil, indicating that the GABA/benzodiazepine receptor complex in the dorsal HP mediates the anxiolytic-like effects of this flavonoid [86]. Interestingly, this same effect on anxiety-like behavior during diestrus was prevented by microinjection of neurosteroid allopregnanolone at 0.5 μg into the dorsal HP, which was blocked by picrotoxin, bicuculline, and flumazenil in the EPM [86]. In contrast, in a surgical menopause model in rats characterized by high anxiety-like behavior associated with a permanent reduction of steroid hormones, chrysin at 2 and 4 mg/kg and diazepam at 1 mg/kg, reversed this anxiety-like behavior, which was blocked by a previous injection of picrotoxin [26]. The fact that picrotoxin, bicuculline, and flumazenil prevented the anxiolytic-like effect of different doses of chrysin supports the idea that its pharmacological effects are established on the GABA/benzodiazepine receptor complex, as occurs with clinically effective GABAergic anxiolytic drugs and several neurosteroids, such as allopregnanolone [87], but does not produce the typical sedative effects of benzodiazepines [24]. However, we cannot discard the possibility of other neurotransmitter systems’ participation and the anti-inflammatory and antioxidant effects in different regions of the brain due to the anxiolytic-like effects of chrysin (Figure 2). Specific studies are required to support or discard this possibility.

## 5. Antidepressant-like Effects of Flavonoid Chrysin

Few studies have explored the antidepressant-like effects of chrysin; however, their results are promising. Filho et al. [18] reported that chrysin at 5 and 20 mg/kg for 28 days increased sucrose consumption and decreased immobility in the tail suspension test (TST) in female mice C57B/6J exposed to CUMS, which is considered to have antidepressant-like effects in pre-clinical research. This effect was also associated with an increase in serotonin, BDNF, and NGF levels, and decreased pro-inflammatory levels of cytokines, such as TNF-α, IFN-γ, IL-1β, and IL-6 in the HP and PFC of C57B/6J mice [11,18]. Additionally, chrysin at 20 mg/kg for 14 days produced an antidepressant-like effect in the FST in male mice C57B/6J subjected to depression induced by olfactory bulbectomy. This effect was associated with decreased pro-inflammatory cytokines (i.e., TNF-α, IFN-γ, IL-1β, IL-6), kynurenine (KYN, a metabolite resulting from serotonin degradation), and indolamine-2, 3-dyoxigenase (IDO, enzyme responsible for serotonin metabolism) activity, besides producing an increase in BDNF and serotonin levels in HP [25]. Interestingly, chrysin at 1, 5, and 10 mg/kg for 28 days produced antidepressant-like effects in the FST in male Wistar rats [13]. In addition, chrysin at 1 and 5 mg/kg for 28 days significantly reduced 5-HT_1A_ receptor expression in the raphe nucleus and increased it in HP, whereas 5-HT_2A_ receptor expression was increased in HP [13]. These effects were similar to those produced by the antidepressant fluoxetine at 1 mg/kg for 28 days. In another study, chrysin at 20 mg/kg for 28 days produced antidepressant-like effects in the TST and FST in female C57BL/6 mice exposed to a depression model induced by hypothyroidism, which was associated with increased serotonin and dopamine levels in the HP [12].

As previously mentioned, a reduced concentration of ovarian hormones in women during their transition to menopause, increases the risk of developing anxiety and depression symptoms [88]. Interestingly, using a surgical menopause model in Wistar rats, it was reported that chrysin at 1 mg/kg reversed depression-like behavior in the FST; this effect was similar to that produced by neurosteroids progesterone at 1 mg/kg and allopregnanolone at 1 mg/kg [37]. The effects of chrysin and neurosteroids were blocked by the previous administration of bicuculline, a selective competitive antagonist of the binding site of γ-aminobutyric acid in the GABA_A_ receptor, which supports the idea that activation of the GABAergic system participates in the antidepressant-like effect of chrysin, as has been reported with neurosteroids [89].

**Figure 3:**
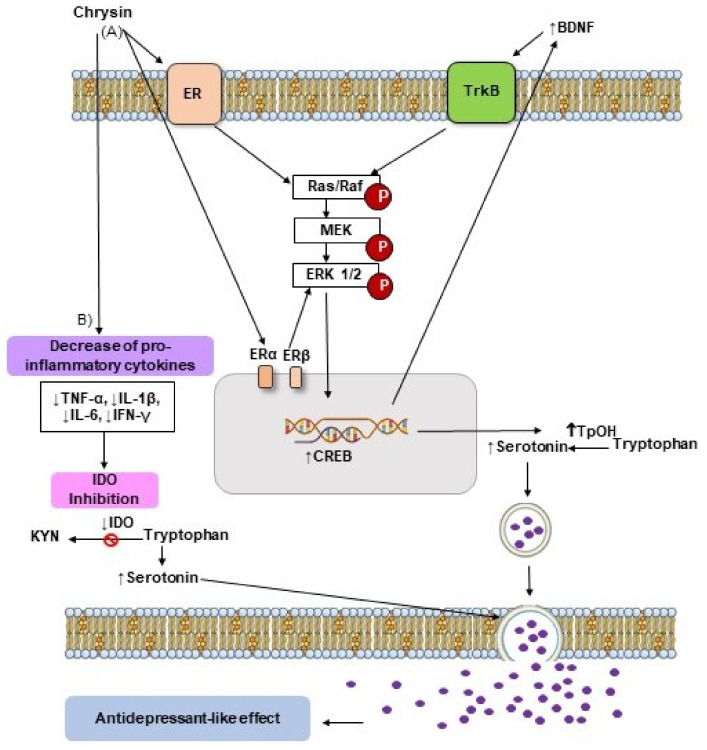
Possible mechanisms of action involved in the antidepressant-like effect of chrysin. (**A**) The flavonoid chrysin can modulate ERα and ERβ of membrane, which triggers the MAPK/ERK1/2 signaling pathway involved in phosphorylation and subsequent CREB activation (↑CREB), which promotes the increase of BDNF levels (↑BDNF) [11,19,20,43], which can further activate the MAPK/ERK1/2 signaling by the TrkB interaction [56]. The above-mentioned pathway also promotes an increase of TpOH expression (↑TpOH) and serotonin levels (↑Serotonin) resulting in the antidepressant-like effect [12,25]. (**B**) Furthermore, chrysin can decrease the pro-inflammatory cytokine levels (↓TNF-α, ↓IL-1β, ↓IL-6, ↓IFN-γ), which inhibits IDO activity (↓IDO) improving serotonergic neurotransmission and producing its antidepressant-like effect [25]. ER = estrogen receptor; MAPK = mitogen-activated-protein-kinases; CREB = cAMP response element binding; BDNF = brain derived neurotrophic factor; TrkB = tropomyosinreceptor kinase B; TpOH = tryptophan-hydroxylase; TNF-α = tumor necrosis factor-α; IL-1β = interleukin 1 beta; IL-6 = interleukin 6; IFN-γ = interferon gamma; IDO = indoleamine 2,3-dioxygenase; ERK1/2 = extracellular signal-regulated kinase 1 and 2; KYN = kynurenine. (Figure was prepared by the authors).

Based on the results described above, we suggest that the mechanism of action underlying the antidepressant-like effect of chrysin involves multiple neurochemical processes, such as the activation of neurotransmitter systems, anti-inflammatory and antioxidant processes, and the activation of neurotrophic factors (Figure 3); however, further exploration is required to improve our understanding of these mechanisms underlying the antidepressant-like effects of chrysin, and to explore its effects in controlled clinical trials.

## 6. Future Considerations

It is noteworthy that human studies focused specifically on the anxiolytic and antidepressant properties of the flavonoid chrysin are nonexistent. Despite this, the results from pre-clinical studies are promising and support the potential therapeutic effects of chrysin, which are similar to those produced by clinically effective anxiolytic and antidepressants drugs. However, unlike other drugs, chrysin does not produce side effects on motor activity associated with sedation. These results from in vitro and in vivo studies in preclinical research support the feasibility to evaluate the potential anxiolytic and antidepressant effects of the flavonoid chrysin in human patients. In the future, it could play a role in developing new pharmacological strategies to ameliorate the symptoms of anxiety and depression disorders in patients nonrespondent to conventional antidepressant and anxiolytic drugs.

For many years, increased monoamine availability in the synaptic cleft has been hypothesized as the mechanism underlying the therapeutic effects of clinically effective antidepressant drugs [90]. A wide range of antidepressant drugs has been developed based on this hypothesis [90]. This is a relevant key that supports the potential antidepressant actions of chrysin, considering that this flavonoid can increase serotoninergic and dopaminergic neurotransmission, which is associated with its antidepressant-like effect [18,25]. In addition, activation of the GABAergic system by this flavonoid supports its potential anxiolytic properties.

Currently, it is known that an increase in BDNF levels is one of the main effects of antidepressant drugs and significantly contributes to their therapeutic effects [91]. Accordingly, a relatively recent meta-analysis found higher serum BDNF concentrations in patients with major depression disorder after 4 to 12 weeks of antidepressant treatment with a selective serotonin reuptake inhibitor and a selective noradrenaline recapture inhibitor [92]. Similarly, treatment with vortioxetine (an inhibitor of the serotonin transporter; 5–15 mg over 4 weeks) increased the plasma BDNF levels in patients with major depression disorder, based on their basal values [93]. This is important considering that chrysin is similar to conventional antidepressants, which increases the concentration of this neurotrophin in pre-clinical research [11].

Neuroinflammation has also been shown to play a crucial role in the risk of neuropsychiatric disorders, including anxiety and depression [58,65]. In agreement with this, some meta-analyses have reported that administration of clinically effective antidepressant drugs, such as selective serotonin reuptake inhibitors, selective noradrenaline reuptake inhibitors, and tricyclic antidepressants drugs, decrease the pro-inflammatory cytokine levels (IL-1β, IL-6, and TNFα) in patients diagnosed with major depression disorder [94,95,96]. Pre-clinical research has also demonstrated that chrysin can reduce pro-inflammatory cytokine levels [11,18,25], which are positively correlated with depression-like behavior. Similarly, oxidative stress has been shown to be involved in the risk of anxiety and depression disorders [46,47], and the antioxidant effects of chrysin identified in pre-clinical research may contribute to its anxiolytic and antidepressant effects.

Finally, as mentioned above, the different neurochemical changes associated with chrysin treatment may play a significant role in the establishment of anxiolytic- and antidepressant-like effects of chrysin, which could be helpful in the treatment of particular groups of patients. Therefore, in future studies, it will be important to explore the anxiolytic and antidepressant effects of chrysin in particular groups of subjects, considering the etiology of anxiety and depression symptoms. This could help identify specific groups of patients in which chrysin could be used as an alternative for the treatment of anxiety and depression disorders, where their etiology could be related to changes in steroid hormones, neurotransmitters, oxidative stress, or neuro-inflammatory processes. It could permit the evaluation of therapeutic effects of chrysin in human patients, as has occurred, for example, with the neurosteroid allopregnanlone [83,87,97,98], and the mechanism of action underlying its anxiolytic and antidepressant effects is shared with those produced by the flavonoid chrysin.

## 7. Conclusions

Chrysin exerts diverse pharmacological effects on the peripheral and CNS. Its action is in particular on some neurotransmitter systems, by activating neurotrophic factors, regulating biomarkers of oxidative stress, and regulating inflammatory and apoptotic signaling pathways, which contributes to the anxiolytic- and antidepressant-like effects of this flavonoid. Although these effects have been evaluated principally in mice and rats, the results are solid and convincing, and could contribute to clinical evaluation of its potential anxiolytic and antidepressant effects in particular groups of patients in a short time. In summary, chrysin is a natural molecule that could become a novel and promising complementary therapy for anxiety and depression disorders in humans.

## Figures and Tables

**Table 1:** Some neurochemical effects produced by flavonoid chrysin potentially involved in its anxiolytic- and antidepressant-like effects.

Activity	Chrysin Treatment	Effects	Reference
Antioxidant	20 mg/kg/30 days, p.o.	↓ TBARS, lipid hydroperoxides, conjugated dienes tissue, circulatory levels ↑ SOD, CAT, GPx, Gsr activity, GSH, GSTs, vitamin C and vitamin E levels in ethanol-induced toxicity in rats	[22]
	50 mg/kg/12 days, i.p.	↑ GHS levels and CAT and SOD activity in heart homogenate in male rats	[27]
	30 and 60 mg/kg/28 days, p.o.	↑ NO and GHS levels, GSHPx, CAT, and SOD activity in rat heart homogenate	[28]
	60 mg/kg/28 days, p.o	↑ 8-OHdG, TBARS levels ↓ GSH, CAT, NO levels	[29]
	1 and 10 mg/kg/60 days, p.o.	↑ SOD, CAT and GPx activity in PFC and HP of aged mice	[20]
	1.25, 2.5, and 5 µM/30 min exposure	↓ ROS formation in neuronal SH-SY5Y and microglial THP-1 cells in vitro	[30]
	10, 30, and 100 mg/kg/44 days, p.o.	Protects against aluminum-induced oxidative stress by restored LPO levels and SOD and CAT activity in cortex and HP of male Swiss mice	[30]
Anti-inflammatory	7.50, 4.75, and 120.90 µM, 18 h exposure	↓ NO, PGE_2_ and TNF-α biosynthesis in CLP-induced RAW 264.7 cells	[31]
	30 mg/kg/2 weeks, i.p.	↓ ALT and AST activity ↓ TNF-α and IL-1β levels ↑ IL-10 and adiponectin in high-fat feeding mice	[32]
	25 and 50 mg/kg/12 days, i.p.	↓ NF-κB, iNOS, COX-2, and TNF-α expression in heart homogenate of DOX-induced cardiotoxicity mice	[27]
	30 and 60 mg/kg/28 days, p.o.	↑ PPAR-γ and TGF-β expression ↓ NF-κBp65 and IKK-β expression and TNF-α level in heart homogenate of isoproterenol-induced myocardial injury rats	[28,29]
	5 and 20 mg/kg/28 days, p.o.	↓ TNF-α, IL-1β and IL-6 levels in PFC and HP of chronically stressed mice	[18]
	5 mg/kg/1 h before LP, i.p.	↓ AST and TNF-α serum levels in septic mice survival	[33]
	5 µM/24 h exposure	↓ iNOS, IL-1β, and TNF-α expression in microglial THP-1 cells exposed to LPS	[30]
GABAergic/BZD	3 µM, 60 min exposure	Acts as competitive ligand for central BZD site in bovine cerebral cortical membranes in vitro	[15]
	13 µM, 60 min exposure	Acts as competitive ligand for peripheral BZD binding site in rat kidneys membranes in vitro	[15]
	1 mg/kg, i.p.	Activates the GABA_A_/BZD receptor complex in male CF1 mice	[24]
	1 mg/kg, i.p.	Activates the GABA_A_/BZD receptor complex in male Sprague Dawley rats	[17]
	0.62 µM, 2 h exposure	Acts as competitive ligand for central BZD site in synaptosomal fractions of rat brain in vitro	[34]
	10 and 30 µM, 30 s exposure	Modulates the activity of Cl^−^ ion channel in the GABA_A_ receptor expressed in Xenopus oocytes in vitro	[35]
	2 mg/kg, i.p.	↓ Anxiety-like behavior by modulating Cl^−^ ion channel in the GABA_A_ receptor of cycling female rats	[36]
	2 mg/kg, i.p.	↓ Depression-like behavior by modulating GABA-binding site in the GABA_A_ receptor of ovariectomized female rats	[37]
	0.5 µg/rat, i.h.	↓ Anxiety-like behavior by modulating GABA_A_/BZD receptor complex in the dorsal hippocampus of cycling female rats	[14]
Serotonergic	5 and 20 mg/kg, p.o.	↑ 5-HT levels and 5-HIAA/5-HT ratio in HP of chronic stressed mice	[18]
	20 mg/kg/28 days, p.o.	↑ 5-HT levels in PFC and HP in female mice with hypothyroidism	[12]
	50 mg/kg twice a day per 4 days, p.o.	↑ 5-HT levels in the striatum of the rat brain	[38]
	10 and 30 mg/kg/2 weeks, p.o.	↑ 5-HT spinal levels ↓ 5-HIAA/5-HT ratio in male mice with experimental neuropathy	[39]
	5 mg/kg/28 days, i.p.	↓ 5-HT_1A_ receptor expression in the dorsal raphe ↑ 5-HT_1A_ and 5-HT_2A_ in the hippocampus of male rats	[13]
Dopaminergic	10 mg/kg/28 days, p.o	↑ DA striatal levels in mice	[19]
	50, 100 and 200 mg/kg/5 days, p.o.	↑ DA levels in striatum of mice treated with 1-methyl-1,2,3,6-tetrahidropidine	[40]
	20 mg/kg/28 days, p.o.	↑ DA levels in PFC and HP in a hypothyroidism model in female mice	[12]
Noradrenergic	50, 100 and 150 mg/kg, i.p.	↓ NE serum levels in rats with pain induced by formalin	[41]
	20 mg/kg/28 days, p.o.	No effects	[12]
Anti-apoptotic	25 and 50 mg/kg/12 days, i.p.	↓ Bax, caspase-3, and cytochrome c activity ↑ Bcl-2 expression in rat heart tissue extract	[27]
	30 and 60 mg/kg/28 days, p.o.	↑ Bcl-2 expression ↓ Bax and caspase-3 activity	[29]
	5 and 20 mg/kg/28 days, p.o.	↓ Caspase-3 and caspase-9 activity in HP and PFC of chronically stressed mice	[18]
	25, 50 and 100 mg/kg/3 days, p.o.	↓ Apoptotic index in cerebral cortex and HP of rats with traumatic brain injury	[42]
Neuroendocrine	5 and 20 mg/kg/28 days, p.o.	↓ Corticosterone plasma levels in chronically stressed mice	[11]
	5 and 20 mg/kg/28 days, p.o.	↓ CRH and ACTH in chronically stressed mice	[18]
	50, 100 and 150 mg/kg, i.p	↓ Corticosterone serum levels in rats with pain induced by formalin	[41]
Neurotrophic	5 and 20 mg/kg/28 days, p.o.	↑ BDNF and NGF levels in PFC and HP in chronically stressed mice	[11]
	1 and 10 mg/kg/60 days, p.o.	↑ BDNF levels in HP and PFC in aged mice	[20]
	10 mg/kg/28 days, p.o.	↑ BDNF and NGF levels in striatum in a Parkinson’s disease model in mice	[19]
	20 mg/kg/28 days, p.o.	↑ BDNF and NGF in HP and PFC in mice subjected to a hypothyroidism model	[43]

TBARS = thiobarbituric acid reactive substance; SOD = superoxide dismutase; CAT = catalase; GPx = glutathione peroxidase; Gsr = glutathione reductase; GSTs = glutathione-S-transferase; GSH = reduced glutathione; NO = nitric oxide; GSHPx = plasma glutathione peroxidase; 8-OHdG = 8-hydroxy-2′-deoxyguanosine; PFC = prefrontal cortex; HP = hippocampus; ROS = reactive oxygen species; LPO = lipid peroxidation; PGE_2_ = prostaglandin E 2; TNF-α = tumor necrosis factor-α; CLP = cecal ligation and puncture procedure; ALT = alanine aminotransferase; AST = aspartate aminotransferase; IL-1β = interleukin 1 beta; IL-10 = interleukin 10; NF-κBp65 = nuclear transcription 116 factor kappa B heterodimer; iNOS = inducible nitric oxide synthase; COX- 2 = cyclooxygenase-2; DOX = doxorubicin; PPAR-γ = peroxisome proliferator-activated receptor-gamma; TGF-β = transforming growth factor-beta; IKK-β = inhibitor of nuclear factor kappa-B kinase subunit beta; IL-6 = interleukin-6; LPS = lipopolysaccharide; BZD = benzodiazepine; GABA = gamma-aminobutyric acid; 5-HT = 5-hydroxytriptamina, serotonin; 5-HIAA = 5-hydroxyindoleacetic; 5-HT_1A_ = serotonin 1A receptor; 5-HT_2A_ = serotonin 2A receptor; DA = dopamine; NE = noradrenaline; BAX = pro-apoptotic protein of the subfamily Bax; Bcl-2 = B-cell lymphoma-2; CRH = corticotropin-releasing hormone; ACTH = adrenocorticotropic hormone; BDNF = brain-derived neurotrophic factor; NGF = nerve growth factor; ↑ = increase; ↓ = decrease; p.o. = per oral route; i.p. = intraperitoneal injection; i.h. = intrahippocampal microinjection. (Table was prepared by the authors).

## Data Availability

Not applicable.
